# Supplementary material for: Fungal secondary metabolism is governed by an RNA-binding protein CsdA/RsdA complex
Source: Nat Commun. 2023 Nov 14;14:7351. doi: 10.1038/s41467-023-43205-2 (PMC10645843; doi:10.1038/s41467-023-43205-2)
Supplement: Supplementary file 1 — Supplementary Information [file 41467_2023_43205_MOESM1_ESM.pdf]

Supplementary information

Fungal secondary metabolism is governed by an RNA-binding protein CsdA/RsdA complex

Zili Song[1,2], Shuang Zhou[1], Hongjiao Zhang[1,2], Nancy P. Keller[3], Berl R. Oakley[4], Xiao Liu[1], Wen-Bing Yin[1,2,*]

[1] State Key Laboratory of Mycology, Institute of Microbiology, Chinese Academy of Sciences, Beijing, 100101, PR China

[2] Savaid Medical School, University of Chinese Academy of Sciences, Beijing, 100049, PR China

[3] Department of Medical Microbiology and Immunology, University of Wisconsin-Madison, Madison, WI, 53706, USA

[4] Department of Molecular Biosciences, University of Kansas, Lawrence, KS, USA

***Corresponding author:** Prof. Dr. Wen-Bing Yin, **Email:** yinwb@im.ac.cn

ORCID: 0000-0002-9184-3198

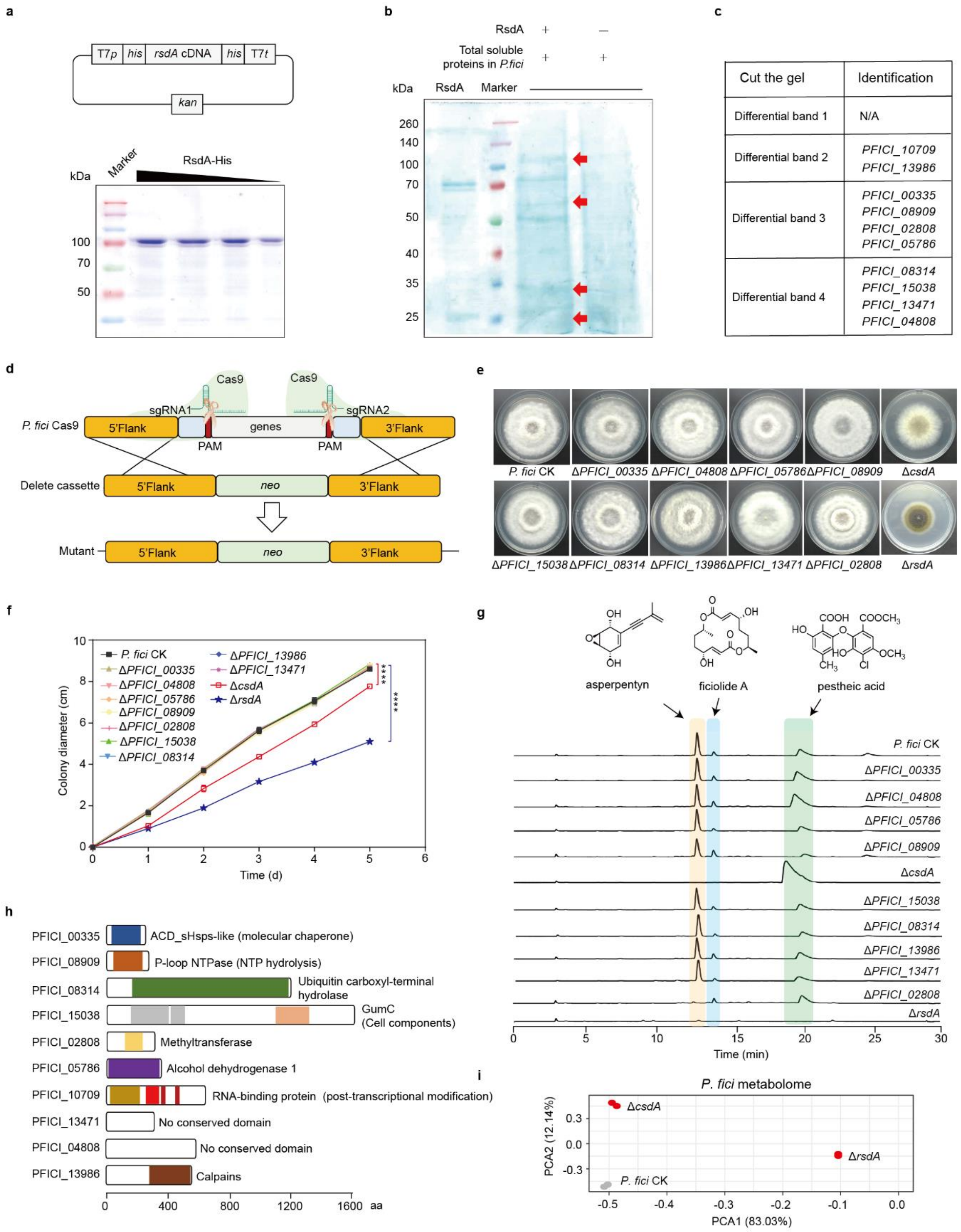
a
T7p his rsdA cDNA his T7t
kan
kDa Marker RsdA-His
100
70
50
b
RsdA + –
Total soluble proteins in P.fici + +
kDa RsdA Marker
260
140
100
70
50
40
35
25
c
Cut the gel | Identification
Differential band 1 | N/A
Differential band 2 | PFICI_10709 PFICI_13986
Differential band 3 | PFICI_00335 PFICI_08909 PFICI_02808 PFICI_05786
Differential band 4 | PFICI_08314 PFICI_15038 PFICI_13471 PFICI_04808
d
Cas9 Cas9
sgRNA1 sgRNA2
P. fici Cas9 5'Flank genes 3'Flank
PAM PAM
Delete cassette 5'Flank neo 3'Flank
Mutant 5'Flank neo 3'Flank
e
P. fici CK ΔPFICI_00335 ΔPFICI_04808 ΔPFICI_05786 ΔPFICI_08909 ΔcsdA
ΔPFICI_15038 ΔPFICI_08314 ΔPFICI_13986 ΔPFICI_13471 ΔPFICI_02808 ΔrsdA
f
P. fici CK
ΔPFICI_00335
ΔPFICI_04808
ΔPFICI_05786
ΔPFICI_08909
ΔPFICI_02808
ΔPFICI_15038
ΔPFICI_08314
ΔPFICI_13986
ΔPFICI_13471
ΔcsdA
ΔrsdA
****
****
Colony diameter (cm)
Time (d)
g
asperpentyn ficiolide A pestheic acid
P. fici CK
ΔPFICI_00335
ΔPFICI_04808
ΔPFICI_05786
ΔPFICI_08909
ΔcsdA
ΔPFICI_15038
ΔPFICI_08314
ΔPFICI_13986
ΔPFICI_13471
ΔPFICI_02808
ΔrsdA
Time (min)
h
PFICI_00335 ACD_sHsps-like (molecular chaperone)
PFICI_08909 P-loop NTPase (NTP hydrolysis)
PFICI_08314 Ubiquitin carboxyl-terminal hydrolase
PFICI_15038 GumC (Cell components)
PFICI_02808 Methyltransferase
PFICI_05786 Alcohol dehydrogenase 1
PFICI_10709 RNA-binding protein (post-transcriptional modification)
PFICI_13471 No conserved domain
PFICI_04808 No conserved domain
PFICI_13986 Calpains
0 400 800 1200 1600 aa
i
P. fici metabolome
ΔcsdA
ΔrsdA
P. fici CK
PCA2 (12.14%)
PCA1 (83.03%)

Supplementary Figure 1. Screening of RsdA interacting proteins from *P. fici*. a, Brilliant blue G-stained 12% SDS–PAGE of RsdA protein. Lane 1: marker. Lane 2-5: different concentrations of His-labeled RsdA protein. **b**, Brilliant blue G-stained 12% SDS–PAGE of the RsdA-associated proteins. Lane 1: purified RsdA protein (80.4 kDa), control group. Lane 2: marker. Lane 3: proteins captured by RsdA from the total proteins of *P. fici*, experimental group. Lane 4: total proteins extracted from *P. fici*, control group. Arrows: differential bands (1, 3-4) cut for LC-MS analysis. **c**, Proteins identified in each differential band. **d**, Schematic of the CRISPR/Cas9 system for gene deletions of ten candidate targets in *P. fici*. **e**, Comparative analysis of colony morphology between ten mutants and Δ*rsdA* mutant of *P. fici*. **f**, Measurement of radial growth in10 mutants and Δ*rsdA* mutant of *P. fici*. $n = 5$ biologically independent replicates. Statistical analysis was performed by using Two-way ANOVA (Significant at ****$p < 0.0001$). **g**, HPLC analysis of SMs in *P. fici* and their mutants. UV absorptions at 254 nm are illustrated. **h**, Conserved domain analysis of 10 target proteins in *P. fici*. **i**, Principal component analysis (PCA) was used to analyses the metabolomic data of Δ*csdA* and Δ*rsdA* mutants. Statistical analysis was performed by using *t* test (two-tailed), and the exact *p* values were shown in Supplementary Data 3 ($n = 2$ biologically independent replicates). Differential metabolic ions: $p < 0.05$, $|\mathrm{Log_2 foldchange}| > 1$. Source data are provided as a Source Data file.

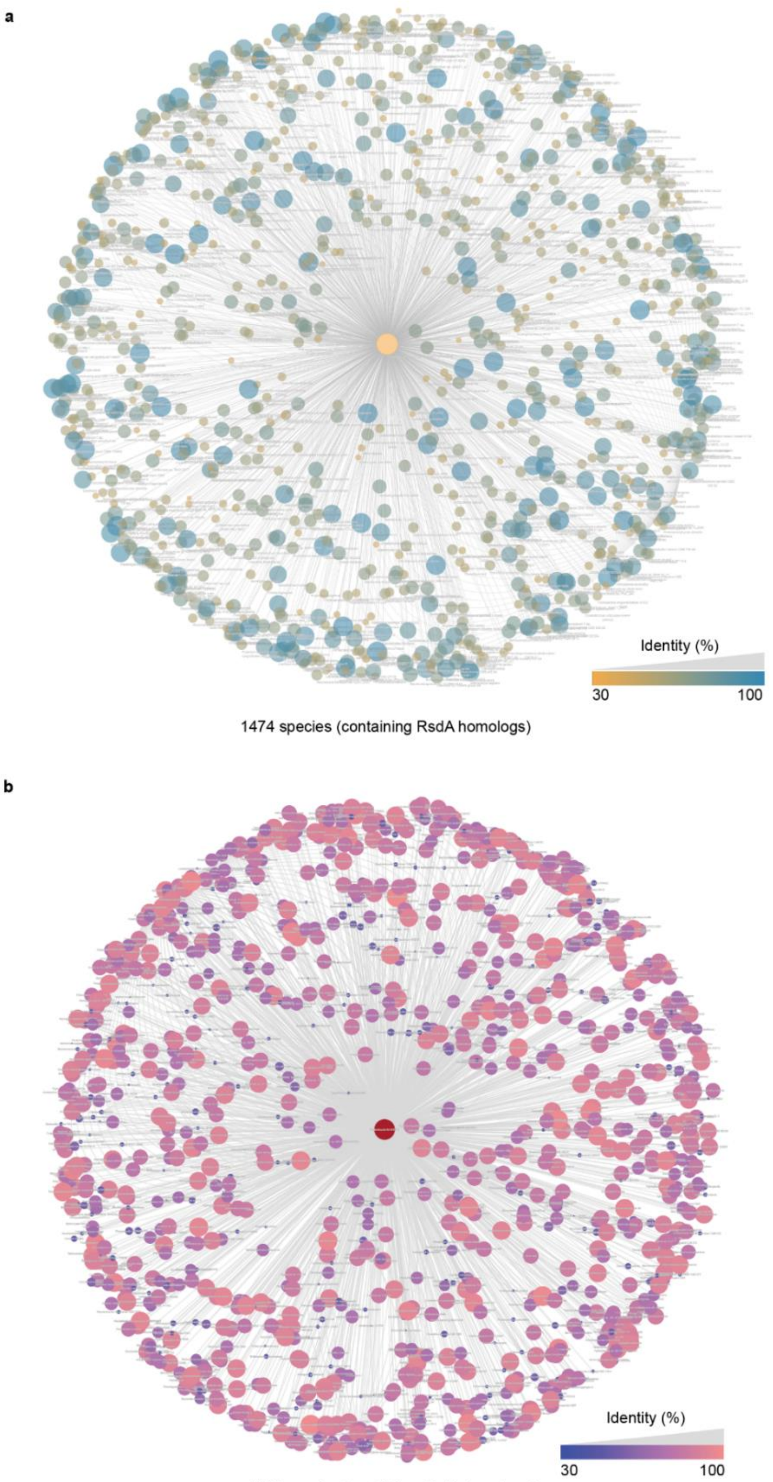
a
Identity (%)
30
100
1474 species (containing RsdA homologs)
b
Identity (%)
30
100
1535 species (containing CsdA homologs)

Supplementary Figure 2. The species containing homologous proteins of RsdA or CsdA in the NCBI database. **a**, 1,474 species containing RsdA homologs. **b**, 1,535 species containing CsdA homologs. The identity and coverage of homologous proteins were more than 30%. Source data are provided as a Source Data file.

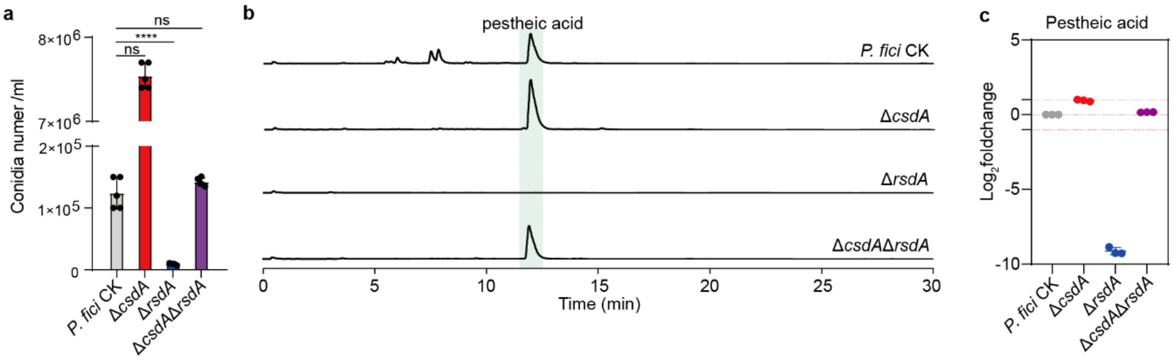

Supplementary Figure 3. Analysis of conidial number and secondary metabolites in the Δ*csdA*Δ*rsdA* mutant of *P. fici*. a, Conidial production in *P. fici* and its mutant strains. Statistical analysis was performed by using One-way ANOVA. “ns”: not significant, p = 0.0787 (Δ*csdA*) or 0.9652 (Δ*csdA*Δ*rsdA*); Significant at ****p < 0.0001. Data are presented as means ± SD (n = 5 biologically independent replicates). **b,** HPLC analysis of SMs in *P. fici* and its mutant strains. UV absorptions at 254 nm are illustrated. **c,** Production of pestheic acid in *P. fici* and its mutant strains. Data are presented as means ± SD (n = 3 biologically independent replicates). Source data are provided as a Source Data file.

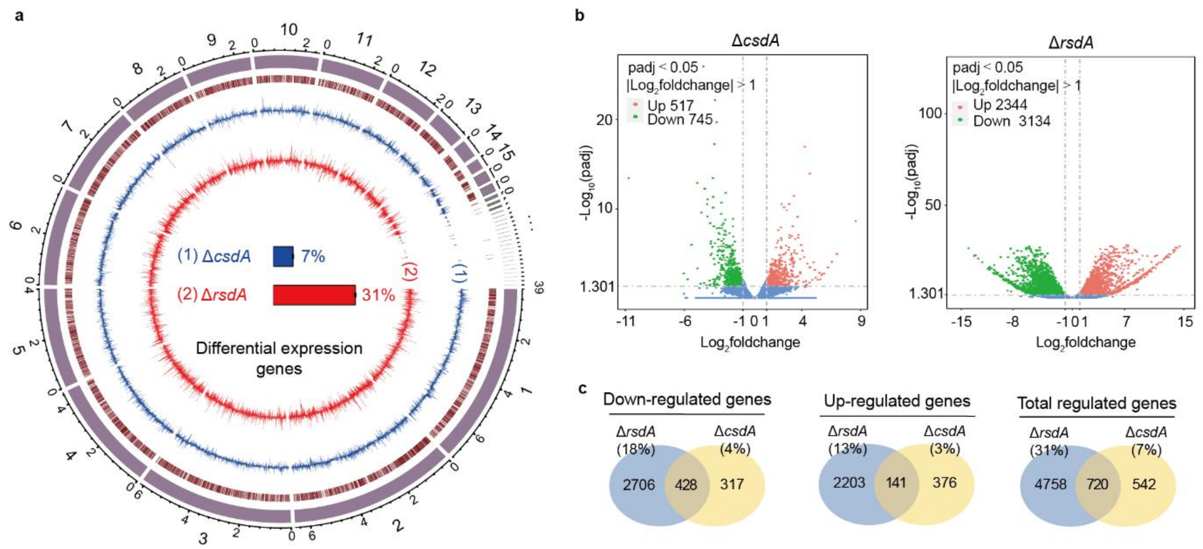

Supplementary Figure 4. Transcriptome analysis of gene expressions regulated by CsdA and RsdA in *P. fici*. a, Differentially expressed genes in the Δ*csdA* and Δ*rsdA* mutants compared with control strain. The "1-39" located outside track represent the scaffolds of *P. fici* genome. The "0, 2, 4, 6" located inside track represents the length of scaffolds. Differentially expressed genes: $p<0.05$, $|\text{Log}_2\text{foldchange}|>1$. **b**, The volcano plots show the differentially expressed genes of Δ*csdA* and Δ*rsdA* mutants versus the control strain. The red dots represent up-regulated genes. The green dots represent down-regulated genes. **c**, Venn analysis of genes co-regulated by CsdA and RsdA in *P. fici*. Statistical analysis was performed by using *t* test (two-tailed), and the exact *p* values were shown in Supplementary Data 4 (n = 3 biologically independent replicates).

a

	Protein	Description	Homologous proteins of *P. fici*	Coverage	Identity	GenBank accession
Developmental regulated genes	StuA	APSES family protein, required for conidiophore formation and morphology	PFICI_06302	94%	46.49%	XP_007833074.1
	MedA	Conidial formation and morphology	PFICI_12799	50%	61.71%	XP_007839571.1
	FlbA	Asexual sporulation and normal colony development	PFICI_04255	70%	60.50%	XP_007831027.1
	FadA	Promote mycelia growth and inhibit asexual development	PFICI_12987	100%	94.05%	XP_007839759.1
	VosA	Velvet-like protein, spore maturation	PFICI_09660	42%	35.78%	XP_007836432.1
	VelB	Conidiation, required for conidia maturation	PFICI_01876	65%	56.69%	XP_007828648.1
Regulators	CsnE	COP9 signalosome subunit 5	PFICI_12631	98%	58.89%	XP_007839403.1
	RlmA	MADS-Box transcription factor	PFICI_06921	79%	57.78%	XP_007833693.1
	VeA	Negative regulator of asexual development	PFICI_11371	43%	47.29%	XP_007838143.1
	LaeA	Sexual development and secondary metabolism regulator	PFICI_06903	73%	38.81%	XP_007833675.1
	HdaA	Histone deacetylase	PFICI_08988	78%	48.54%	XP_007835760.1
	Esa-1	Histone acetyltransferase	PFICI_12848	100%	71.91%	XP_007839620.1

b

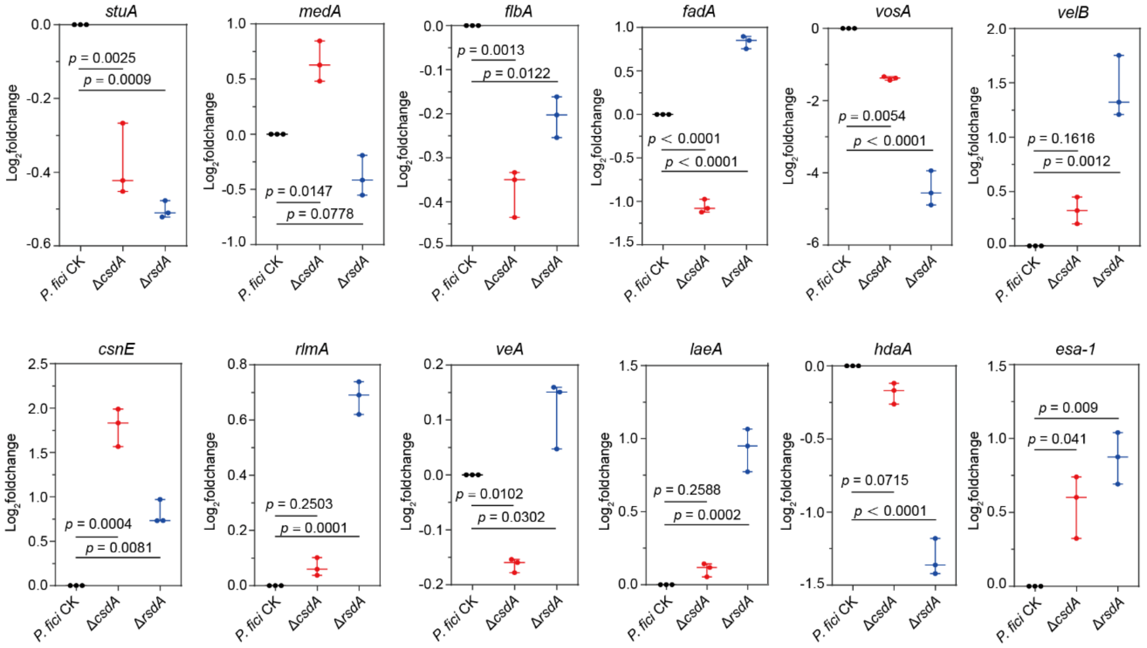

Supplementary Figure 5. Transcriptome analysis of developmental genes regulated by CsdA and RsdA. a, The development-related genes in *P. fici* were identified by blastp. **b** and **c**, Transcriptome analysis of the effects of CsdA and RsdA on development and regulatory genes in *P. fici*. Statistical analysis was performed by using *t* test (two-tailed), and the exact *p* values were shown in (**b** and **c**). Data are presented as means ± SD (n = 3 biologically independent replicates). Source data are provided as a Source Data file.

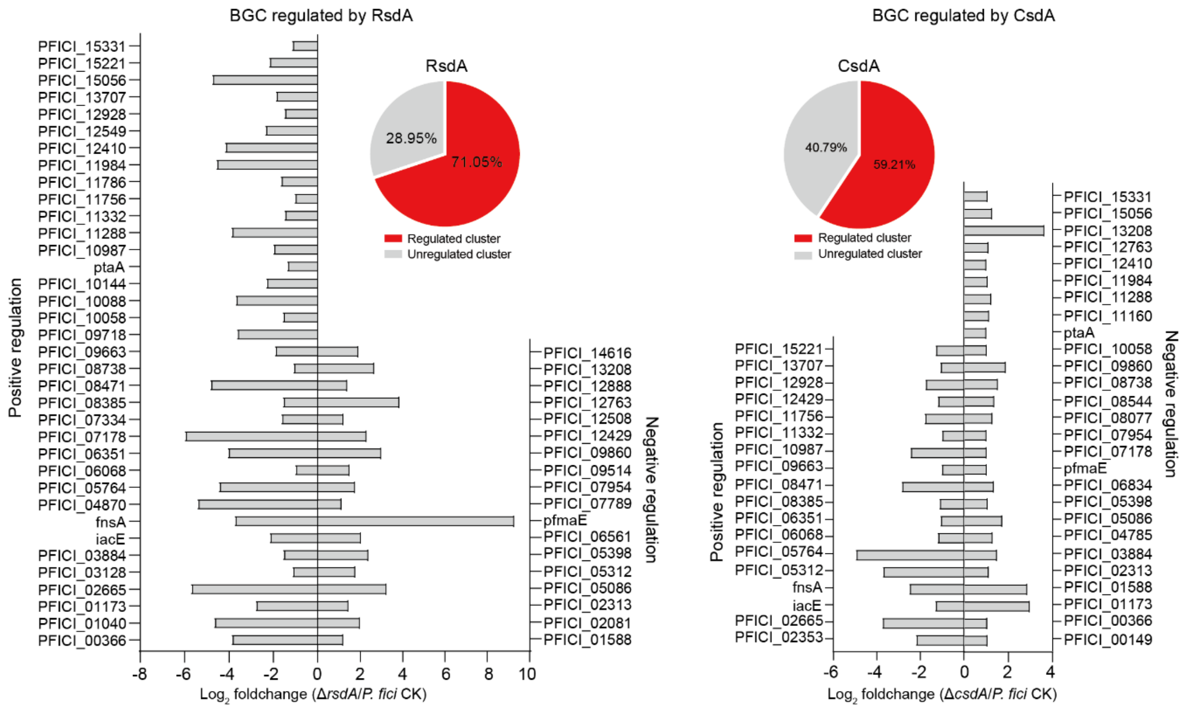

Supplementary Figure 6. Secondary metabolism related genes are regulated by CsdA or RsdA. Significantly regulated BGC backbone genes were shown. Statistical analysis was performed by using *t* test (two-tailed), and the exact *p* values were shown in Supplementary Data 4 (n = 3 biologically independent replicates). Differentially expressed genes: $p < 0.05$, $|Log_2foldchange| > 1$. Source data are provided as a Source Data file.

a

RsdA (730 aa)

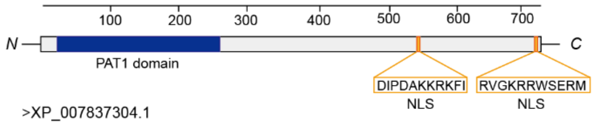

>XP_007837304.1

MVVASMQHHPHHSQGPAPPQHLQHPRPSSIVHQQHHQAPPPQSQHPSAYSSYQPAGQT
QGSQHGHAQEGLPYYAHPSPYSTPGTTSGYPAADTSDMMAAAQMPRPYPPIYHTPQSNS
PASVASPSGHDQHRSLYGAAAPSIQQQQSMYYSAQQPYSSMPQQAAPSPYQQHAQQPQ
HSMTSQPNMMMSHPGSQHPIQQHTNQHAQQGLNSPRHTKIETQVPPQLQRPASGPLGTP
QHNQNGSGPLSTPGGSSPGVNQNAAPGPIPATTPLVVRQDNNGVQWIAFEYSRDRVKME
YTIRCDVESVNTDELPSDFKTENCVYPRACCPKDQYRGNRLQYETECNTVGWALAQLNPS
LRGKRGLIQRAVDSWRNSNQDPRLRSRRVRRMAKMTNRKAVQTPHPSHMSGPSGPAGIP
APGAMGPGGGTAMGKPGMSNMGSHMHHHQGHSDGNSQGGEEVGDGEYMDEGHHHH
HQAPSGPGSGHGDDVRQAQVFSGYPQPYPGTSASGPGMPHSSHHNAIAARHSTRDDDE
APEGLFPDIPDAKKRKFILVDDNKRGGRLRVRVTLENVDTKEIPDSFRKSSSVFPRSYFPRE
MQSPPPSATGSRFFENDLEDDDVEVEGGRTGRSRAAGRVMVKVPFSDGSEDQIAIPRTRK
STRGKEVRLNDLGYRMAWLQSRVFSGRTIFLQRALDCYRNKTRNAIESVMQDVKTTAPHY
ETRVGKRRWSERMRQSTDKRDDE

Euk-mPLoc 2.0 computation result

Query protein	Predicted location (s)
XP_007837304.1	Nucleus

NLS Mapper

Sequence	Score
DIPDAKKRKFI	8.5
RVGKRRWSERM	5.5

b

CsdA (634 aa)

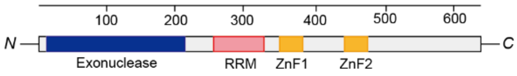

>XP_007837481.1

MSTVPPQPPHFDRYVVIHVATTCDEHGVYVTKDSAEVIELGWIELDAKSLEELHRESVLV
KPINTPITPLCTSLTTLTWEHVRNAGTFRDAINRFDAFASEHLTNQNLDFVFVTLDAWDLR
VQLPREARDKAVVLPPYLQHSRTFDLRTEYQRWQQHHPESLPFGPSMLSNICAALEVEP
VQSSAPIKHNLPFHLQALAPASPRRAMEEAITLARVLRGLIRKSQPAHEHPDVLTRPMDA
RADVRAFLSERSKVLHMSGLPHDTTQSELESWFTQFGGRPIAFWTLRTPEQHKPTGTGF
AVFSSHEEAAESLCMNGRALNEKAIEVSPSSSRVLDRAAEILTPFPPSKNRPRPGDWTC
PSCGFSNFQRRTACFRCSFPAVGAGGPQNEMSSYGGGGGGSGGYGYGAPPMMPPPH
HGGHHHHHGGMGHGGGRMGGSGVVPFRAGDWKCGNEVCGYHNFAKNVCCLRCGAS
RAGAAVVADSGYPSPMDSGSNYGMGSHSVGGTPGPGPYASAGGPFGGAGGGYGQHF
GGPPSTYALPSGIGGGAAPYPSALNTHIGGPAPGSHSAGPFDSRAAEAAFQSAGNGPA
SAGPSNNFFGSQAENDPFAFLSTGIGGLTVSGDARQNGSGAGGPQSKSPAA

Euk-mPLoc 2.0 computation result

Query protein	Predicted location (s)
XP_007837481.1	Extracell, nucleus

NLS Mapper

Sequence	Score
No	No

c

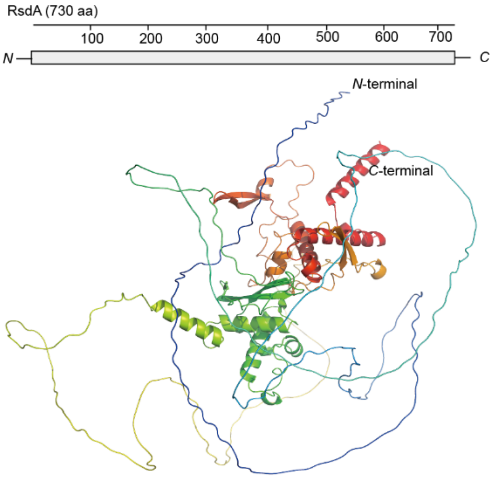

d

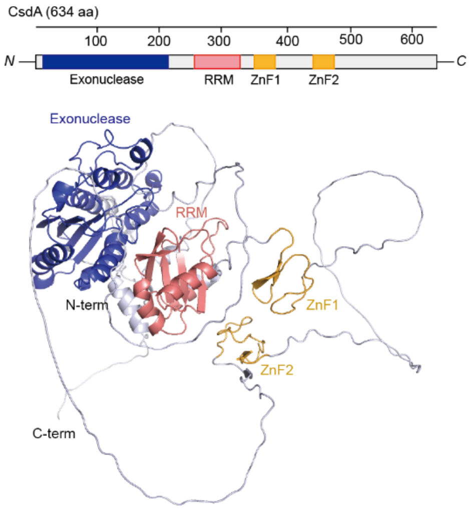

74

Supplementary Figure 7. The prediction of the subcellular localization and protein structure of CsdA and RsdA in *P. fici*. a and **b**, Prediction of subcellular localization of RsdA and CsdA in *P. fici* by Euk-mPLoc2 and NLS Mapper. The *C*-terminal of RsdA has two nuclear localization signals (NLS), but CsdA does not have. **c** and **d**, The predicted three-dimensional structure of RsdA and CsdA protein obtained by AlphaFold2. "*N*-term": *N*-terminal, the starting site for protein synthesis. "*C*-term": *C*-terminal, the termination site of protein synthesis. "Exonuclease": DEDDh 3'-5' exonuclease. "RRM": RNA recognition motif. "ZnF": Zn-finger. "β": β-sheet. "α": α-helix. RRM contains four antiparallel β-sheets arranged in the order β4-β1-β3-β2 and two superimposed α-helices, while the two ZnF are separated.

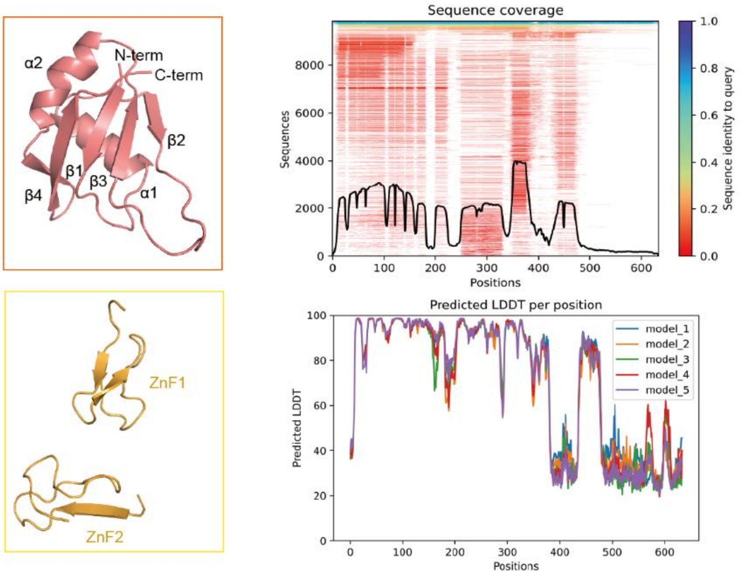

Supplementary Figure 8. The score of CsdA protein structure predicted by AlphaFold2. LDDT, local distance different test. The prediction structure with the highest score of CsdA was used for topology analysis.

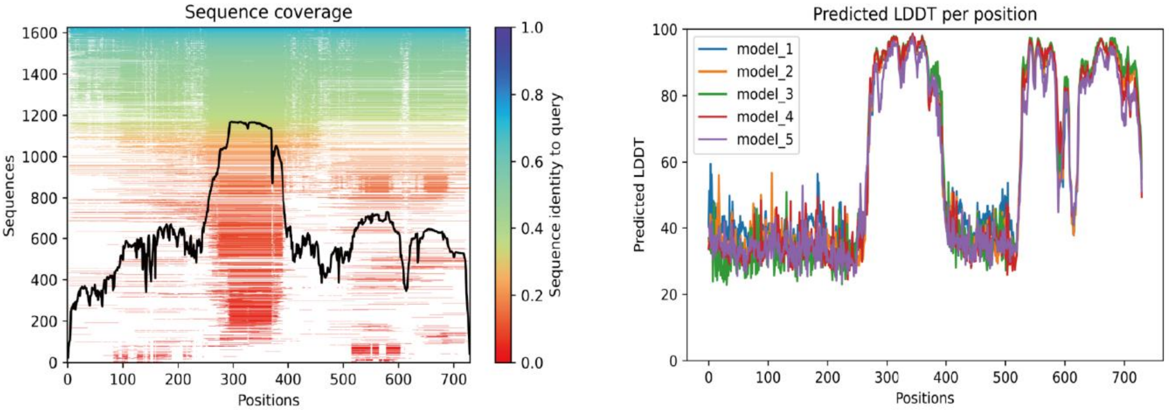

Supplementary Figure 9. The score of RsdA protein structure predicted by AlphaFold2. LDDT, local distance different test. The prediction structure with the highest score of RsdA was used for topology analysis.

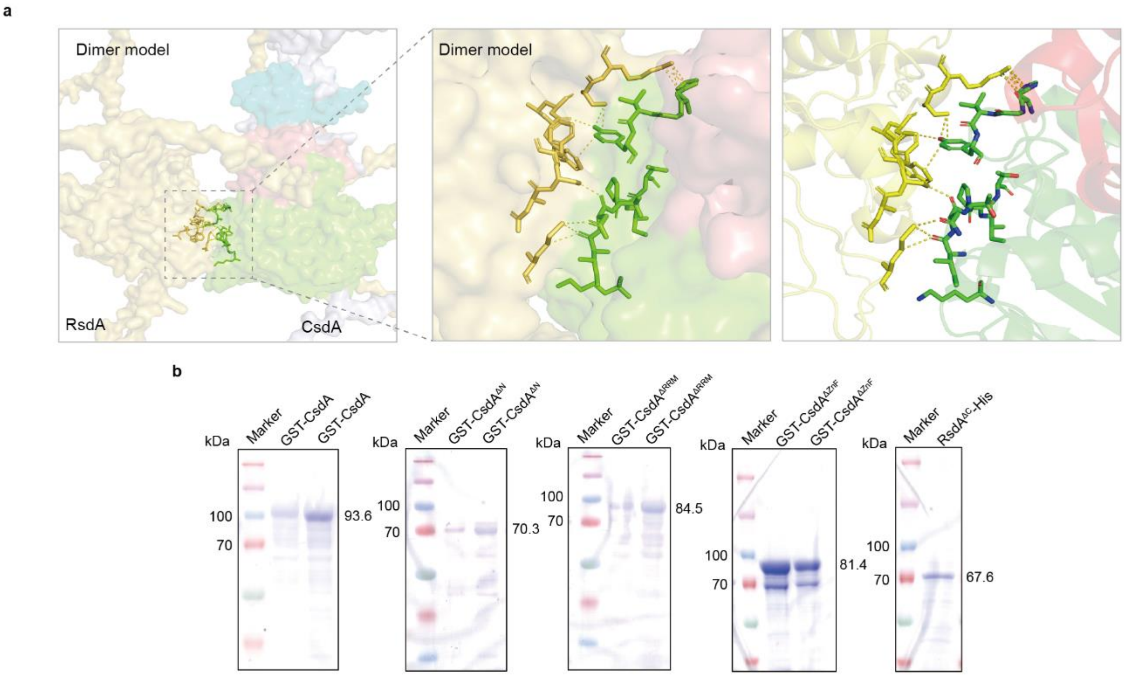

Supplementary Figure 10. Prediction of CsdA/RsdA dimer model and purification of CsdA or RsdA related proteins. a, The dimer structure of CsdA and RsdA was predicted by Alphafold2. The docking sites on the RsdA amino acid sequence are shown at the *C*-terminal sites S668 and R669. The docking sites of the CsdA amino acid sequence are shown in the *N*-terminal exonuclease domain. Yellow represents the protein model of RsdA. Green, red, and cyan represent exonuclease, RRM, and ZnF domains of CsdA, respectively. **b**, The expression and purification of CsdA or RsdA related proteins in *E. coli* BL21. Glutathione S-transferase (GST) was fused to the *N*-terminal of CsdA and its associated proteins (GST-CsdA$^{\Delta N\text{-terminal}}$, GST-CsdA$^{\Delta RRM}$, GST-CsdA$^{\Delta ZnF}$), His-tag was fused to both sides of RsdA$^{\Delta C\text{-terminal}}$, and they were detected by 12% SDS PAGE.

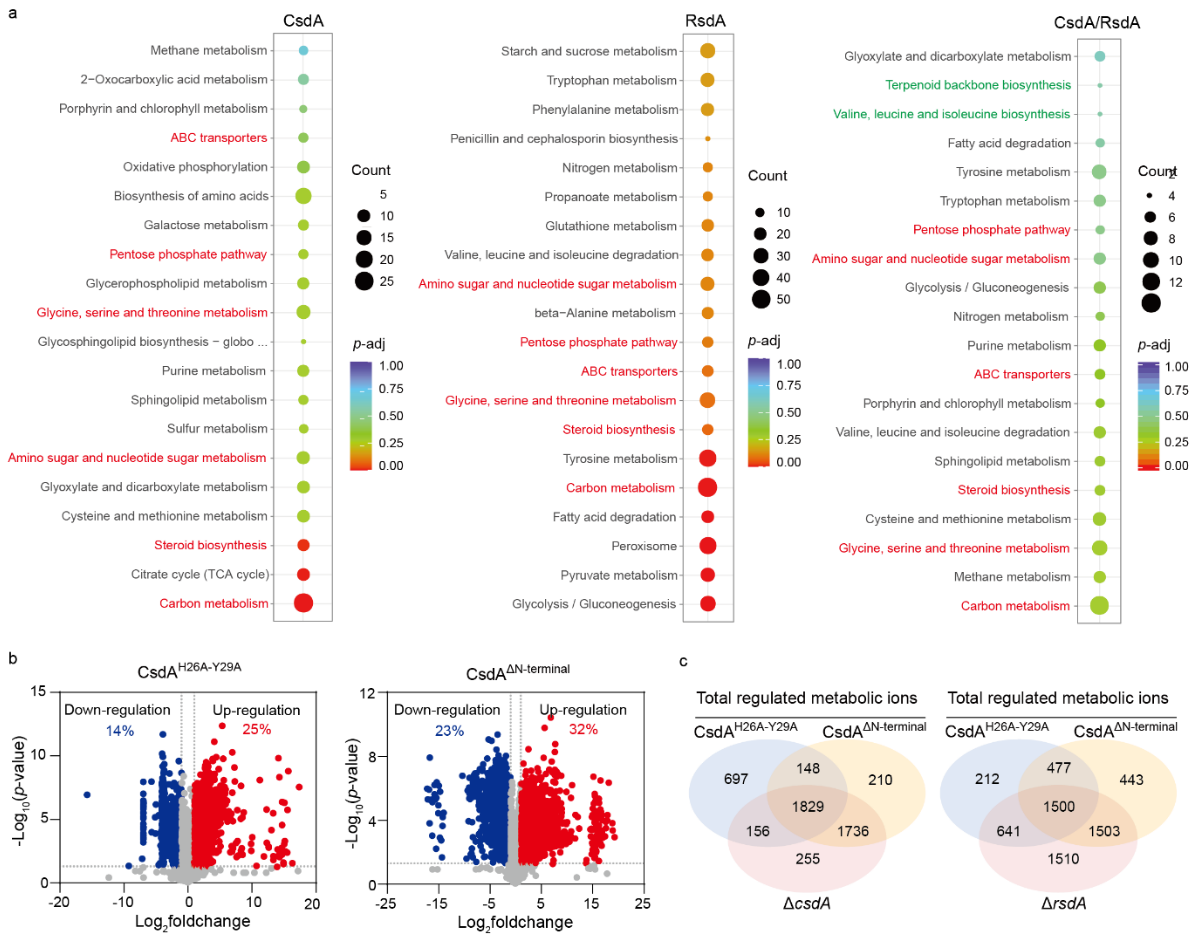

Supplementary Figure 11. The individual functions of CsdA and RsdA and their complex were evaluated by DEGs and metabolome data analysis. a, KEGG was used to analyze the metabolic pathways of CsdA and RsdA respectively and their co-regulated genes. The red fonts represent metabolic pathways involved in CsdA and RsdA and the complex. Cyan represents metabolic pathways individually involved in the complex. Carbon metabolism pathway is the main metabolic pathway involved in all three. **b**, Volcano plots show the differentially regulated metabolic ion peaks of $CsdA^{H26A\text{-}Y29A}$ or $CsdA^{\Delta N\text{-terminal}}$ mutant versus the control. **c**, Distribution of total metabolic ion peaks due to mutations in CsdA protein interaction sites or regions in *P. fici*. Statistical analysis was performed by using *t* test (two-tailed), and the exact *p* values were shown in Supplementary Data 3, 4 (n = 3 biologically independent replicates).

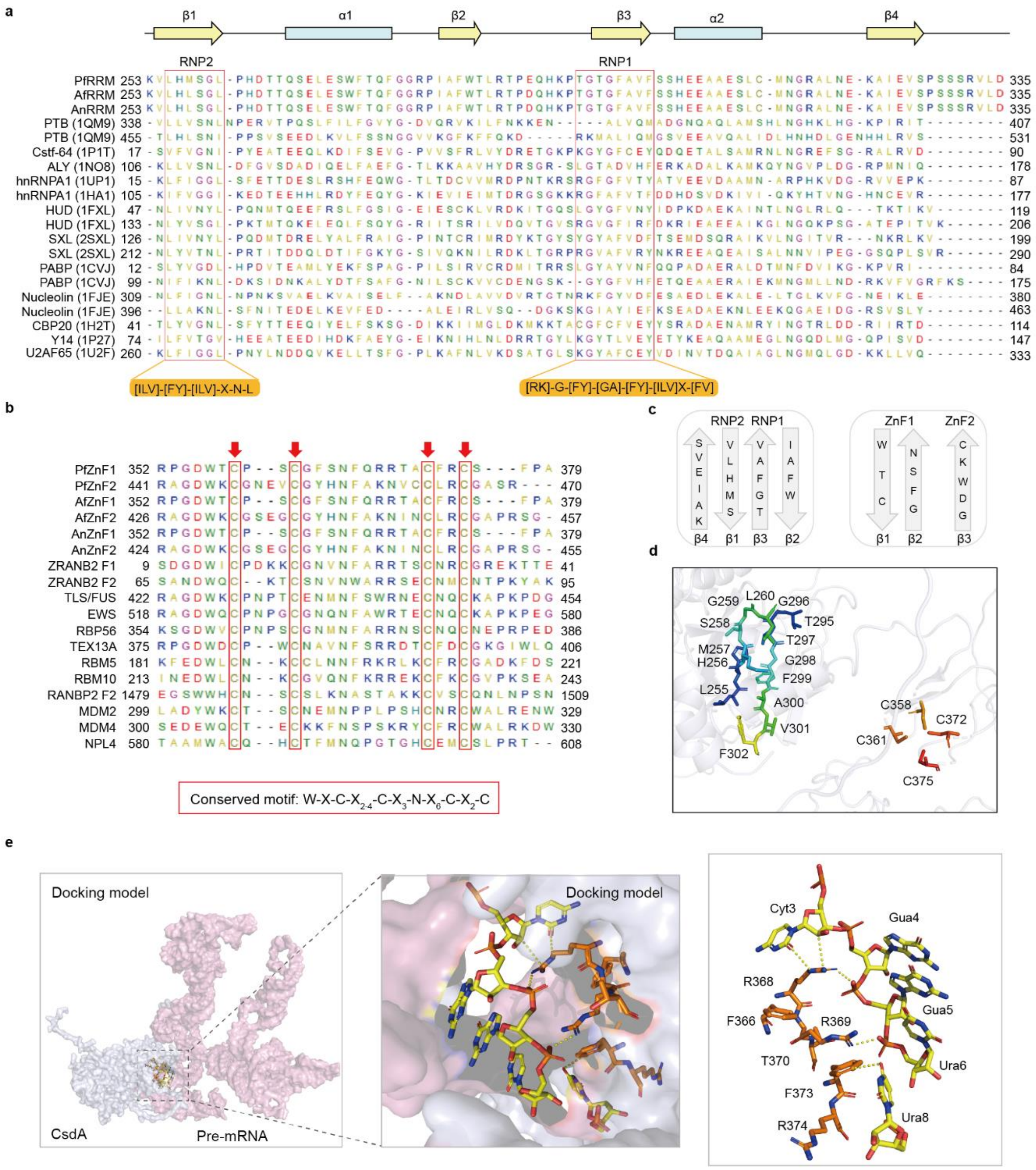
a
β1
α1
β2
β3
α2
β4
RNP2
RNP1
PfRRM
AfRRM
AnRRM
PTB (1QM9)
Cstf-64 (1P1T)
ALY (1NO8)
hnRNPA1 (1UP1)
hnRNPA1 (1HA1)
HUD (1FXL)
SXL (2SXL)
PABP (1CVJ)
Nucleolin (1FJE)
CBP20 (1H2T)
Y14 (1P27)
U2AF65 (1U2F)
[ILV]-[FY]-[ILV]-X-N-L
[RK]-G-[FY]-[GA]-[FY]-[ILV]X-[FV]
b
PfZnF1
PfZnF2
AfZnF1
AfZnF2
AnZnF1
AnZnF2
ZRANB2 F1
ZRANB2 F2
TLS/FUS
EWS
RBP56
TEX13A
RBM5
RBM10
RANBP2 F2
MDM2
MDM4
NPL4
Conserved motif: W-X-C-X2-4-C-X3-N-X6-C-X2-C
c
RNP2 RNP1
ZnF1
ZnF2
β4 β1 β3 β2
β1 β2 β3
d
G259 L260 G296
S258
T295
T297
M257
H256
G298
F299
L255
A300
C358
C372
C361
V301
F302
C375
e
Docking model
CsdA
Pre-mRNA
Docking model
Cyt3
Gua4
R368
Gua5
F366
R369
T370
Ura6
F373
Ura8
R374

Supplementary Figure 12. Conserved motif of CsdA and molecular docking with *rsdA* pre-mRNA. **a**, Multi-sequence alignment of RRM domain in CsdA of *P. fici*. "β": β-sheet. "α": α-helix. The red box represents the ribonucleoprotein complex (RNP). AfRRM: RRM domain in CsdA of *A. fumigatus*. AnRRM: RRM domain in CsdA of *A. nidulans*. Other RRM domains (PTB, Cstf-64, ALY, hnRNPA1, HUD, SXL, PABP, Nucleolin, CBP20, Y14, and U2AF65) have been reported to come from the humans and participate in post-transcriptional modifications related to pre-mRNA recognition and splicing. **b**, Multi-sequence alignment of PfZNF domain in CsdA. The arrows and red boxes represent conserved cysteine residues. AfZnF: ZnF domain in CsdA of *A. fumigatus*. AnZnF, ZnF domain in CsdA of *A. nidulans*. Other ZnF domains (ZRANB2, TLS/FUS, EWS, RBP56, TEX13A, RBM5, RBM10, RANBP2, MDM2, MDM4, NPL4) have been reported to come from the humans and participate in alternative splicing of pre-mRNA. **c**, Schematic representation of amino acid residues in β-sheets of RRM and ZnF domains. "RNP": ribonucleoprotein. Conserved RNP1 and RNP2 were located on β3- and β1-sheet, respectively. **d**, Conserved active sites in PfRRM and PfZnF. **e**, Model of *rsdA* pre-mRNA docking with CsdA. The docking sites on *rsdA* pre-mRNA showed CGGUAU. The docking sites on the CsdA amino acid sequence showed R368, R369, F373.

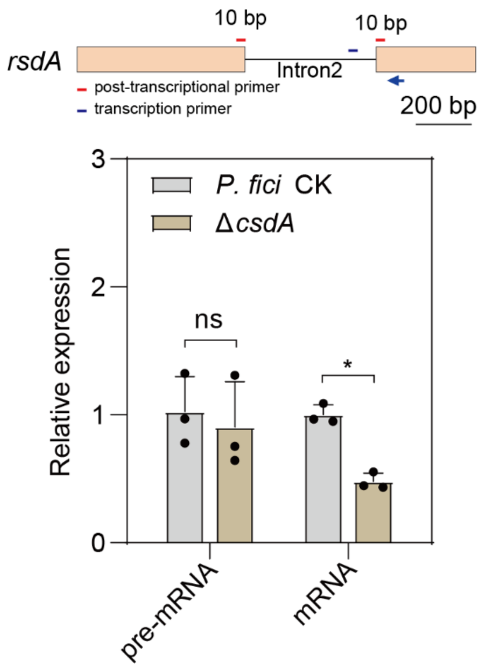

Supplementary Figure 13. Relative abundance of *rsdA* intron 2 at transcriptional and transcriptional levels in Δ*csdA* mutant compared with the control. "pre-mRNA" represents the transcriptional level and "mRNA" represents the post-transcriptional level. The red dashes in the diagram represent the primers designed to amplify regions composed of exon-exon junctions after intron removal (mRNA), while the blue dashes represent the primers designed within the introns that remain unspliced (pre-mRNA). Data are presented as means ± SD (n = 3 biologically independent replicates). Statistical analysis was performed by using Two-way ANOVA ("ns": not significant, p = 0.7865. Significant at *p = 0.0476). Source data are provided as a Source Data file.

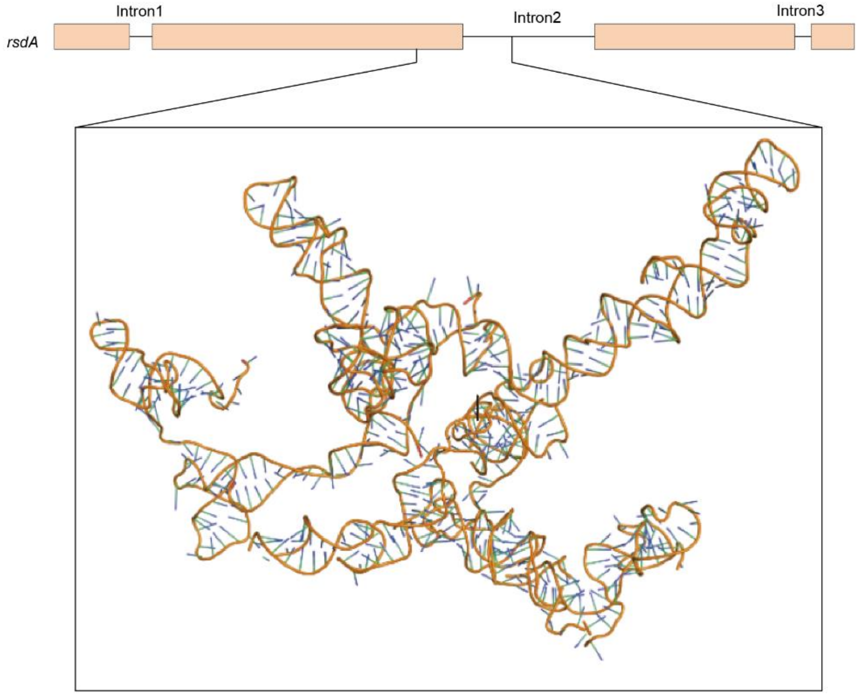

Supplementary Figure 14. The structure of partial *rsdA* pre-mRNA sequence was predicted by 3DRNA software.

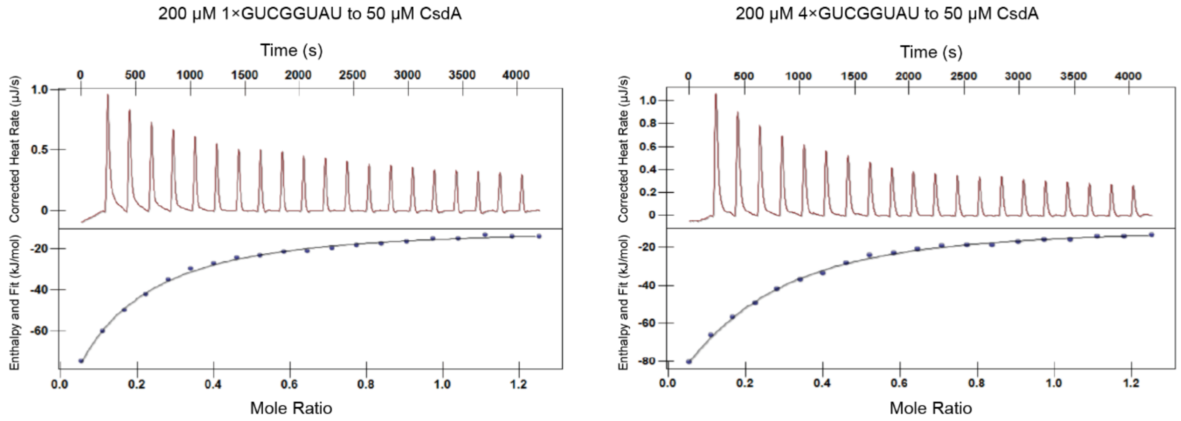

Supplementary Figure 15. The binding of CsdA to GUCGGUAU was determined by isothermal titration calorimeter method. At the left, 200 μM RNA (1×GUCGGUAU) was dribbled into a 50 μM CsdA protein, K_d=19.74±3.67 μM. At the right, 200 μM RNA (4×GUCGGUAU) was dribbled into a 50 μM CsdA protein, K_d=10.48±5.12 μM.

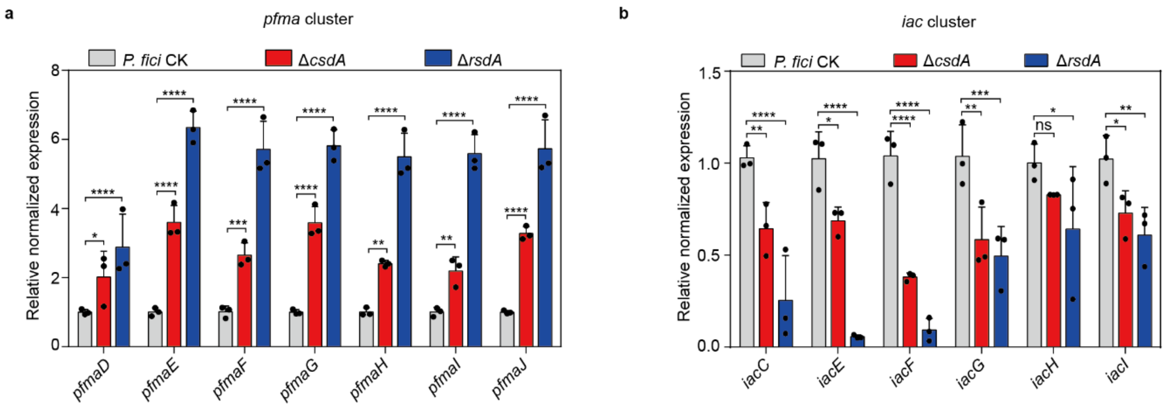

Supplementary Figure 16. The expression level of representative secondary metabolite cluster genes regulated by CsdA and RsdA in *P. fici*. a, Validation of gene expression levels within *pfma* cluster in *P. fici* and their mutants by qRT-PCR. **b**, Validation of gene expression levels within *iac* cluster in *P. fici* and their mutants by qRT-PCR. Data are presented as means ± SD (n = 3 biologically independent replicates). Statistical analysis was performed by using Two-way ANOVA. Significant at *p = 0.026, **p = 0.0019 (*pfmaH*) or 0.0087 (*pfmaI*), ***p = 0.0003 (**a**). “ns”: not significant, p = 0.2726; Significant at *p = 0.0166 (*iacE*), 0.0102 (*iacH*), or 0.0387 (*iacI*), **p = 0.006 (*iacC*), 0.0014 (*iacG*), or 0.0033 (*iacI*), ***p = 0.0002 (*iacG*), ****p < 0.0001 (**b**). Source data are provided as a Source Data file.

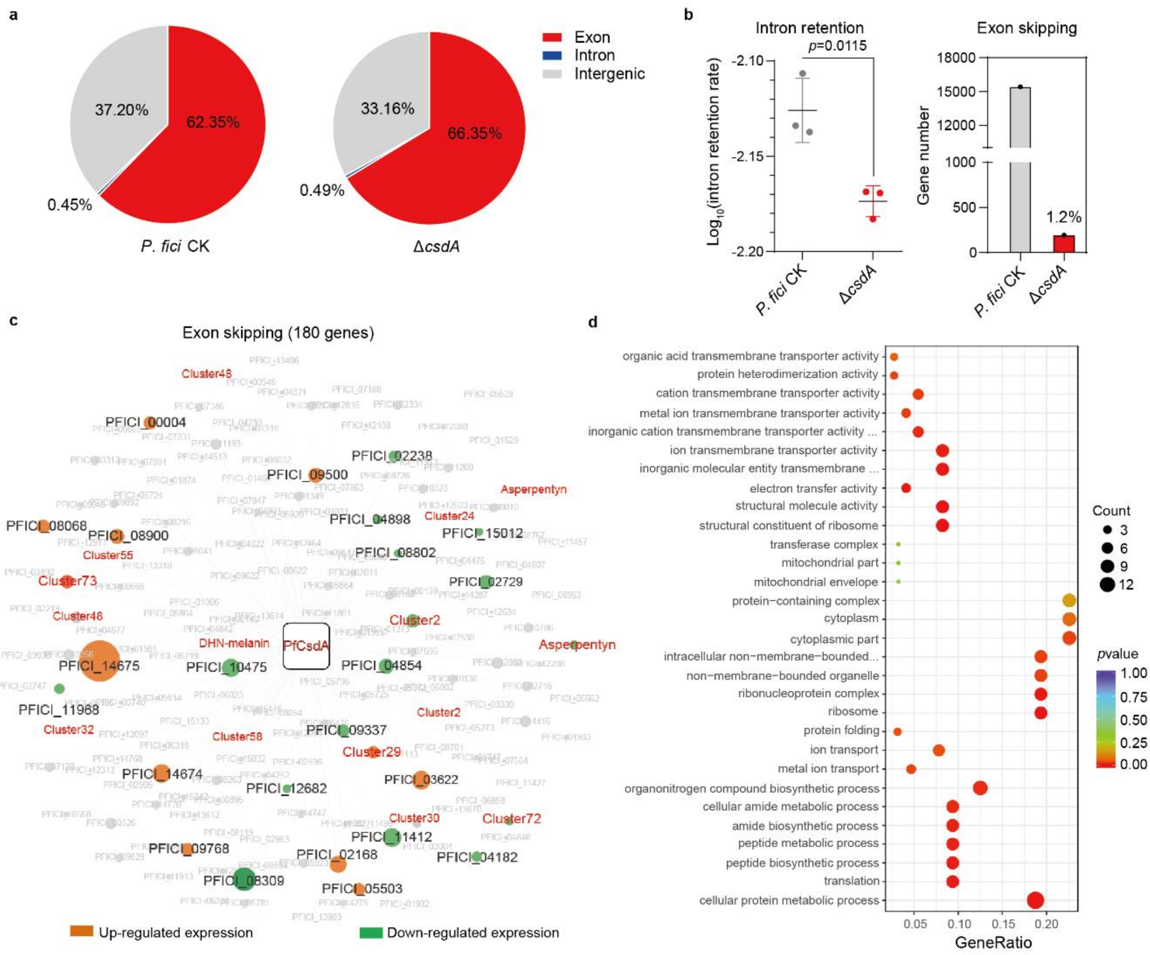

Supplementary Figure 17. Transcriptomic analysis of alternative splicing genes mediated by CsdA in *P. fici*. a, Distribution of total introns and exons in the transcriptome of control strain and Δ*csdA* mutant in *P. fici*. **b**, Alternative splicing events in Δ*csdA* mutant compared with control strain in *P. fici*. Data are presented as means ± SD (n = 3 biologically independent replicates). Statistical analysis was performed by using Two-way ANOVA (Significant at p = 0.0115). **c**, Gene for exon skipping events in Δ*csdA* mutant. Orange represents genes that were significantly up-regulated and green represents genes that were significantly down-regulated (|Log_2foldchange|>1). **d**, GO enrichment analysis of exon skipping genes. Source data are provided as a Source Data file.
