# Supplementary material for: Fungal secondary metabolism is governed by an RNA-binding protein CsdA/RsdA complex
Source: Nat Commun. 2023 Nov 14;14:7351. doi: 10.1038/s41467-023-43205-2 (PMC10645843; doi:10.1038/s41467-023-43205-2)
Supplement: Supplementary file 3 — Description of Additional Supplementary Files [file 41467_2023_43205_MOESM3_ESM.pdf]

Description of Additional Supplementary Files

Supplementary Data 1

Description: Mass spectrometry data of protein identifications. Sheet 1: Total proteins identified by mass spectrometry in a pull-down experiment. Sheet 2: RsdA and other non-specific binding proteins identified by mass spectrometry. Sheet 3: The total proteins of *P. fici* was identified. Sheet 4: Mass spectrum information of 10 candidate proteins. Sheet 5: Mass spectrum information of 201 potential RsdA interacting proteins.

Supplementary Data 2

Description: Species information used to construct phylogenetic tree. Sheet 1: Species used to construct phylogenetic tree. Sheet 2: Proteins used to construct phylogenetic tree.

Supplementary Data 3

Description: High resolution mass spectrometry data of metabolites in *P. fici*. Sheet 1: Total metabolic ion products detected in *P. fici* and its mutants (n = 2 biologically independent replicates). Sheet 2: Total metabolic ionic products co-regulated by CsdA and RsdA (n = 2 biologically independent replicates). Sheet 3: Metabolic ionic products co-upregulated by CsdA and RsdA (n = 2 biologically independent replicates). Sheet 4: Metabolic ionic products down-regulated by CsdA and RsdA (n = 2 biologically independent replicates). Sheet 5: Total metabolic ion products detected in *P. fici* and $CsdA^{H26A\text{-}Y29A}$ mutant (n = 3 biologically independent replicates). Sheet 6: Total metabolic ion products detected in *P. fici* and $CsdA^{\Delta N\text{-terminal}}$ mutant (n = 3 biologically independent replicates). Statistical analysis was performed by using t test (two-tailed), and the exact p values were shown in Excel file. Differential metabolic ions: $p < 0.05$, $|\text{Log}_2\text{foldchange}| > 1$.

Supplementary Data 4

Description: RNA-seq data for regulated BGCs in *P. fici*. Sheet 1: RNA-seq data for regulated BGCs in Δ*rsdA* mutant. Sheet 2: RNA-seq data for regulated BGCs in Δ*csdA* mutant. Sheet 3: RNA-seq data for inversely regulated BGCs in Δ*csdA* and Δ*rsdA* mutants. Sheet 4: RNA-seq data for co-upregulated BGCs in Δ*csdA* and Δ*rsdA* mutants. Sheet 5: RNA-seq data for down-regulated BGCs in Δ*csdA* and Δ*rsdA* mutants. Statistical analysis was performed by using t test (two-tailed), and the exact p values were shown in Supplementary Data 4 (n = 3 biologically independent replicates). Differential metabolic ions: $p < 0.05$, $|\text{Log}_2\text{foldchange}| > 1$.

Supplementary Data 5

Description: High resolution mass spectrometry data of metabolites in *A. fumigatus*. Sheet 1: Total metabolic ion products detected in *A. fumigatus* and its mutants (n = 2 biologically independent replicates). Statistical analysis was performed by using t test (two-tailed), and the exact p values were shown in Excel file. Differential metabolic ions: $p < 0.05$, $|\text{Log}_2\text{foldchange}| > 1$.

Supplementary Data 6
Description: Strains used in this study.

Supplementary Data 7
Description: Plasmids used in this study.

Supplementary Data 8
Description: Primers used in this study.
