# Supplementary material for: Fungal secondary metabolism is governed by an RNA-binding protein CsdA/RsdA complex
Source: Nat Commun. 2023 Nov 14;14:7351. doi: 10.1038/s41467-023-43205-2 (PMC10645843; doi:10.1038/s41467-023-43205-2)
Supplement: Supplementary file 12 — Reporting Summary [file 41467_2023_43205_MOESM12_ESM.pdf]

nature portfolio

Corresponding author(s): Wen-Bing Yin

Last updated by author(s): Oct 25, 2023

Reporting Summary

Nature Portfolio wishes to improve the reproducibility of the work that we publish. This form provides structure for consistency and transparency in reporting. For further information on Nature Portfolio policies, see our Editorial Policies and the Editorial Policy Checklist.

Statistics

For all statistical analyses, confirm that the following items are present in the figure legend, table legend, main text, or Methods section.

n/a	Confirmed	
☐	☒	The exact sample size (*n*) for each experimental group/condition, given as a discrete number and unit of measurement
☐	☒	A statement on whether measurements were taken from distinct samples or whether the same sample was measured repeatedly
☐	☒	The statistical test(s) used AND whether they are one- or two-sided<br>*Only common tests should be described solely by name; describe more complex techniques in the Methods section.*
☒	☐	A description of all covariates tested
☒	☐	A description of any assumptions or corrections, such as tests of normality and adjustment for multiple comparisons
☐	☒	A full description of the statistical parameters including central tendency (e.g. means) or other basic estimates (e.g. regression coefficient) AND variation (e.g. standard deviation) or associated estimates of uncertainty (e.g. confidence intervals)
☐	☒	For null hypothesis testing, the test statistic (e.g. *F*, *t*, *r*) with confidence intervals, effect sizes, degrees of freedom and *P* value noted<br>*Give P values as exact values whenever suitable.*
☒	☐	For Bayesian analysis, information on the choice of priors and Markov chain Monte Carlo settings
☒	☐	For hierarchical and complex designs, identification of the appropriate level for tests and full reporting of outcomes
☒	☐	Estimates of effect sizes (e.g. Cohen's *d*, Pearson's *r*), indicating how they were calculated

Our web collection on statistics for biologists contains articles on many of the points above.

Software and code

Policy information about availability of computer code

Data collection	Waters HPLC system e2695, Mascot website (2.7), Illumina NovaSeq 6000, Agilent HPLC 1200 system, Agilent Masshunter software (B.06.00), Zeiss Axioplan 2 imaging system (version 2), Real-Time PCR Detection System (CFX96), Thermal Cycler (C1000), Nano-Drop (C2000), Isothermal Titration Calorimetry (AFFINITY ITC LV).
Data analysis	antiSMASH (version 7.0), MEGA (version 7.0 and 6.06), RAxML (version 1.3.1), IMAGEJ2 (version 2.15.0), PyMOL (version 2.5), GraphPad (version 8), CRISPR design tool (version 5.4), Sequence Matrix (version 1.7.8), RAxML (version 1.3.1), iTOL (version v6), fastp (version 0.19.7), HISAT2 (version 2.1.0), Integrative Genomics Viewer (version 2.12.3), DESeq2 (version 1.20.0), XCMS software (Online version), OmicStudio tools (version 3.6), Alphafold2 (version 2), 3DRNA (version v2.0), HADDOCK (version 2.4), Launch Nano Analyze software (version 3.11.0).

For manuscripts utilizing custom algorithms or software that are central to the research but not yet described in published literature, software must be made available to editors and reviewers. We strongly encourage code deposition in a community repository (e.g. GitHub). See the Nature Portfolio guidelines for submitting code & software for further information.

Data

Policy information about availability of data

All manuscripts must include a data availability statement. This statement should provide the following information, where applicable:

- Accession codes, unique identifiers, or web links for publicly available datasets
- A description of any restrictions on data availability
- For clinical datasets or third party data, please ensure that the statement adheres to our policy

RNA-seq data generated in this study have been deposited in the Gene Expression Omnibus (GEO) database under accession code GSE241019 [https://www.ncbi.nlm.nih.gov/geo/query/acc.cgi?acc=GSE241019]. Metabolomics data generated in this study have been deposited in the MassIVE database under accession code MSV000091220 [https://massive.ucsd.edu/ProteoSAFe/dataset.jsp?accession=MSV000091220]. The data supporting the findings of the present study are available within the paper and its Supplementary Information. All relevant data, including further image and processed data, are available in Source Data. All DNA and amino acid sequences used in this study are available in the NCBI database [https://www.ncbi.nlm.nih.gov/]. All reported protein or domain structures used in this study are available in the PDB database [https://www.rcsb.org/]. Source data are provided with this paper.

Research involving human participants, their data, or biological material

Policy information about studies with human participants or human data. See also policy information about sex, gender (identity/presentation), and sexual orientation and race, ethnicity and racism.

Reporting on sex and gender	Not applicable.
Reporting on race, ethnicity, or other socially relevant groupings	Not applicable.
Population characteristics	Not applicable.
Recruitment	Not applicable.
Ethics oversight	Not applicable.

Note that full information on the approval of the study protocol must also be provided in the manuscript.

Field-specific reporting

Please select the one below that is the best fit for your research. If you are not sure, read the appropriate sections before making your selection.

☒ Life sciences ☐ Behavioural & social sciences ☐ Ecological, evolutionary & environmental sciences

For a reference copy of the document with all sections, see nature.com/documents/nr-reporting-summary-flat.pdf

Life sciences study design

All studies must disclose on these points even when the disclosure is negative.

Sample size	No statistical methods were used to predetermine sample size. Quantitative assays were performed in three biological triplicates and mean and standard deviation values calculated to ensure reproducibility. At least three independent biological replicates to meet the minimum requirements for statistical analysis.<br>The ITC experiment performed two independent biological replications with good reproducibility, following de facto standards in the field. So we believe our sample sizes were appropriate.
Data exclusions	No data were excluded from the manuscript.
Replication	Reproducibility was verified by performing two or three independent biological replicates and noted. All attempts of replication were successful.
Randomization	Randomization was not appropriate for this study, and the experiments described in this study do not involve random allocation of samples.
Blinding	No blinding was involved during this study as it does not involve animal or human subjects or group allocation.

Reporting for specific materials, systems and methods

We require information from authors about some types of materials, experimental systems and methods used in many studies. Here, indicate whether each material, system or method listed is relevant to your study. If you are not sure if a list item applies to your research, read the appropriate section before selecting a response.

Materials & experimental systems

n/a	Involved in the study
☐	☒ Antibodies
☒	☐ Eukaryotic cell lines
☒	☐ Palaeontology and archaeology
☒	☐ Animals and other organisms
☒	☐ Clinical data
☒	☐ Dual use research of concern
☒	☐ Plants

Methods

n/a	Involved in the study
☒	☐ ChIP-seq
☒	☐ Flow cytometry
☒	☐ MRI-based neuroimaging

Antibodies

Antibodies used

All antibodies used in the study are commercially available, including His-Tag Mouse Monoclonal Antibody (66005-1; RRID: AB_11232599; PROTEINTECH, CHICAGO, USA), GST-Tag Mouse Monoclonal Antibody (66001-2; RRID: AB_2881488; PROTEINTECH, CHICAGO, USA), HRP-Goat Anti-Mouse IgG (H+L) (SA00001-1; RRID: AB_2722565; PROTEINTECH, CHICAGO, USA).

Validation

All antibodies used in this study were certified and validated by manufacturers, as shown below:
His-Tag Mouse Monoclonal Antibody, https://www.ptglab.com/products/His-Tag-Antibody-66005-1-Ig.htm;
GST-Tag Mouse Monoclonal Antibody, https://www.ptglab.com/products/GST-Tag-Antibody-66001-2-Ig.htm;
HRP-Goat Anti-Mouse IgG (H+L), https://www.ptglab.com/products/HRP-conjugated-Affinipure-Goat-Anti-Mouse-IgG-H-L-secondary-antibody.htm.
No newly developed or unvalidated antibodies were included in the manuscript.
